# Diffuse red-brown papules in a toddler

**DOI:** 10.1016/j.jdcr.2026.02.020

**Published:** 2026-02-17

**Authors:** Celine H. Phong, Eli Kasheri, Bonnie A. Lee, Janellen Smith

**Affiliations:** Department of Dermatology, University of California, Irvine, Irvine, California

**Keywords:** benign cephalic histiocytosis, facial papules, histiocytic disorders, non-Langerhans cell histiocytosis, pediatric dermatology

## Case description

A 14-month-old boy presented with a 9-month history of progressive, asymptomatic red-brown skin lesions. The eruption began on the face at age 3 months before spreading to the neck, trunk, and extremities. The patient had not undergone any prior treatments and was otherwise healthy and up to date on vaccinations.

**Fig 1:**
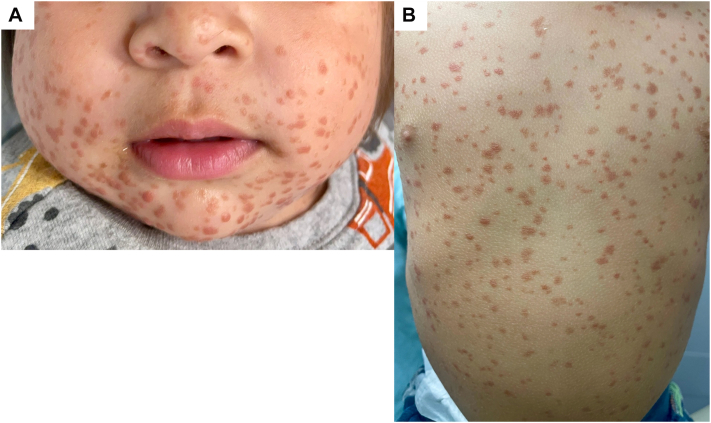
Multiple red-brown macules and smooth flat-topped papules on the face **(A)** and trunk **(B)** in an infant.

**Fig 2:**
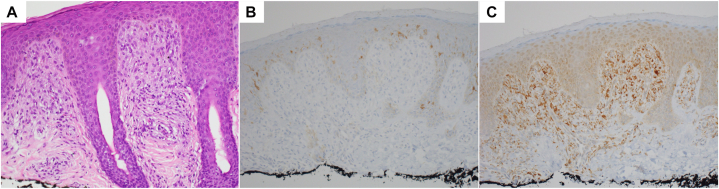
Histopathology showing dense proliferation of enlarged mononuclear cells within the superficial dermis on hematoxylin and eosin staining (**A**, 20×), negative CD1a immunostaining (**B**, 20×), and positive CD68 immunostaining (**C**, 20×)

Physical exam revealed Fitzpatrick skin type III with hundreds of 3-4 mm red-brown macules and smooth, flat-topped papules diffusely distributed (Fig 1). There was no involvement of the oral mucosa. Laboratory evaluation (complete blood count and comprehensive metabolic panel) was unremarkable, though platelets were slightly decreased (197 × 10^3^/μL) and albumin/globulin ratio was elevated. A skeletal survey showed no lytic lesions. A shave biopsy of the anterior thigh demonstrated a dense superficial dermal proliferation of mononuclear cells with abundant cytoplasm. Immunohistochemistry was positive for CD68 and negative for CD1a, S100, and CD117 (c-KIT) (Fig 2).

## Question: What is the diagnosis?

**A.** Diffuse juvenile xanthogranuloma
**B.** Maculopapular cutaneous mastocytosis (formerly urticaria pigmentosa)
**C.** Benign cephalic histiocytosis
**D.** Langerhans cell histiocytosis

## Discussion

The clinical and histologic findings support a diagnosis of benign cephalic histiocytosis (BCH). BCH is a rare non-Langerhans cell histiocytosis (LCH) of early childhood, with approximately 60 cases reported in the English literature. It typically presents as 2-5 mm pink to red-brown papules on the face, occasionally involving the trunk and limbs. Unlike other histiocytoses, BCH is usually limited to the skin, sparing mucous membranes and visceral organs, though 1 case of associated diabetes insipidus has been reported.^1^ BCH generally resolves spontaneously over several years, leaving transient post-inflammatory hyperpigmentation.

The differential diagnosis includes cutaneous mastocytosis, LCH, juvenile xanthogranuloma, and generalized eruptive histiocytoma. Mastocytosis typically spares the central face and is CD117+ on immunohistochemistry. LCH is differentiated by S100 and CD1a positivity. BCH, juvenile xanthogranuloma, and generalized eruptive histiocytoma may exist on a clinical spectrum. While juvenile xanthogranuloma also affects the head and neck, it is frequently associated with café-au-lait macules or neurofibromatosis type 1. Generalized eruptive histiocytoma lesions typically favor the trunk and proximal extremities rather than the face.

The pathogenesis of BCH remains unclear but may involve mutations in the converging PI3K/AKT/mTOR and RAS/RAF/MEK/ERK pathways which have been found in other LCH disorders.^2^ One reported case has shown improvement of BCH with topical sirolimus (a mammalian target of rapamycin inhibitor) suggesting that similar pathways may be overactivated in BCH.^3^ This patient’s mother declined topical treatment with sirolimus as the patient was asymptomatic. On follow-up exam at age 28 months, the papules on our patient’s face had started to flatten and fade without treatment.

This case represents a challenging clinical presentation that may easily be misdiagnosed, as was our patient, who had been previously treated for mastocytosis by an outside provider. However, on initial presentation we felt that his facial involvement was uncharacteristic and further investigation was warranted. This case reminds us how the clinical distribution of lesions can provide important clues to diagnosis in rare dermatologic conditions such as BCH and how ultimately, correlation with histopathology was necessary to clinch the diagnosis.

## Conflicts of interest

None disclosed.

## References

[1] Weston W.L., Travers S.H., Mierau G.W., Heasley D., Fitzpatrick J. (2000). Benign cephalic histiocytosis with diabetes insipidus. Pediatr Dermatol.

[2] Emile J.F., Diamond E.L., Hélias-Rodzewicz Z. (2014). Recurrent RAS and PIK3CA mutations in Erdheim-Chester disease. Blood.

[3] Habeshian K., Silverman R.A., DeKlotz C.M.C. (2019). Treatment of benign cephalic histiocytosis with topical 1% rapamycin ointment. Pediatr Dermatol.

